# Recent Applications of Fluorescence Recovery after Photobleaching (FRAP) to Membrane Bio-Macromolecules

**DOI:** 10.3390/s100605927

**Published:** 2010-06-10

**Authors:** Gamal Rayan, Jean-Erik Guet, Nicolas Taulier, Frederic Pincet, Wladimir Urbach

**Affiliations:** Laboratoire de Physique Statistique de l’Ecole Normale Supérieure, associe aux Universites Paris 6 et Paris 7, CNRS UMR 8550, 24 rue Lhomond, 75005 Paris, France; E-Mails: jean-erik.guet@lps.ens.fr (J-E.G.); nicolas.taulier@upmc.fr (N.T.); pincet@lps.ens.fr (F.P.); urbach@lps.ens.fr (W.U.)

**Keywords:** biomembranes, Fluorescence Recovery after Photobleaching (FRAP), diffusion, biomolecular interactions

## Abstract

This review examines some recent applications of fluorescence recovery after photobleaching (FRAP) to biopolymers, while mainly focusing on membrane protein studies. Initially, we discuss the lateral diffusion of membrane proteins, as measured by FRAP. Then, we talk about the use of FRAP to probe interactions between membrane proteins by obtaining fundamental information such as geometry and stoichiometry of the interacting complex. Afterwards, we discuss some applications of FRAP at the cellular level as well as the level of organisms. We conclude by comparing diffusion coefficients obtained by FRAP and several other alternative methods.

## 1. Introduction

The cellular milieu is dynamic, wherein its constituents undergo fluctuations/motions driven by thermal energy, as well as those induced by active cellular processes. The ubiquitous Singer-Nicolson Fluid Mosaic Membrane Model highlighted the hitherto unemphasized dynamic/fluid nature of the biological membrane composed of lipids and proteins [1]. It is worth mentioning that the first experimental evidence showing the biological membrane is a bilayer was obtained as early as 1925 by Gorter and Grendel, whose eloquent experiment demonstrated that the area of a monolayer of lipids from red blood cells (of several mammals) was twice the surface area of the corresponding intact cells [2]. Their results were rather fortuitous, as they did not consider the membrane surface pressure but nonetheless mark a turning point in the development of membrane models. It would take exactly another 10 years, until a membrane model incorporating proteins would be published by Danielli and Davson [3]. Even though their model was erroneous, with proteins forming a layer on the outside surface of the membrane, it was the first time that proteins were included in the structure of biological membranes [3]. While several models have been proposed essentially as “upgrades” of the Singer-Nicolson Fluid Mosaic Membrane Model, we would like to briefly mention the Mattress Model proposed by Mouritsen and Bloom in 1984 [4]. This model introduces the concept of hydrophobic mismatch (or hydrophobic matching) whereby the lipid bilayer either stretches or compacts in order to match the length of the hydrophobic region of the protein and thus prevent the energetically unfavourable exposure of the protein’s hydrophobic core to the aqueous environment, and it can give rise to clustering of membrane proteins [4]. That hydrophobic matching plays an important biological role (by affecting the activity of membrane proteins) has been demonstrated experimentally even before the advent of the principle. In 1981 (three years prior to the proposal of the Mattress Model) Johannsonn and colleagues [5], and Caffrey and Feigenson [6] published studies which reported that the activity of the sarcoplasmic reticulum (SR) Ca^2+^ ATPase was membrane-thickness dependent. More recently, Dumas *et al.* [7] have illustrated that the optimal activity of melibiose permiase (MelB) occurred when the hydrophobic thickness of the protein matched that of the bilayer. Cornelius [8] has shown that the activity of Na,K-ATPase is strongly influenced by this effect, with deviations from it resulting in decreased protein’s activity and that the addition of cholesterol, which acts by thickening membranes, resulted in the shift of activity towards membranes of shorter lipid lengths. In addition to membrane protein activity, the hydrophobic matching is involved in the sorting of membrane proteins. Munro [9] has eloquently demonstrated that the hydrophobic length of a protein plays an important role in its retention in the Golgi apparatus, whereby those membrane proteins with a smaller hydrophobic/transmembrane domain lengths were more selectively retained in the Golgi apparatus as opposed to the plasma membrane (which retained those proteins containing greater transmembrane domain lengths). Readers are referred to a recent review [10] for further information on the hydrophobic mismatch and its relation to membrane protein function. Additionally, several excellent membrane biophysics books are recommended for further perusal [11–14].

**Figure 1.**
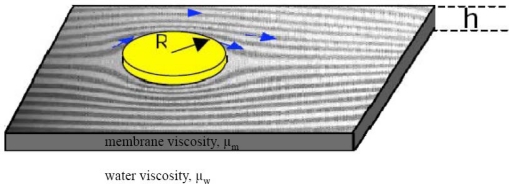
A schematic representation of an object diffusing inside a liquid membrane (after Figure 1 from Saffman and Delbrück [19]).

Membrane proteins are important structural constituents of the cell membrane, and additionally play diverse roles in the function of the cell. There are two kinds of motions that membrane proteins can undergo; rotational and lateral diffusion. The former refers to the rotation of proteins around the axis that is perpendicular to the plane of the membrane, while the latter refers to the diffusion within the membrane. Since the focus of this review is lateral diffusion of membrane proteins (and to a lesser extent of other biopolymers), those interested in rotational diffusion of membrane proteins are advised to read the following references [15–17]. Protein diffusion within cells played an instrumental role in the development of the Fluid Mosaic Membrane Model, particularly the experiments of Frye and Edidin, who fused human and murine cells and observed the intermixing of different membrane proteins that was caused by their diffusion within the (fused) membrane [18]. As we shall see later, lateral diffusion of proteins can provide valuable information on the state of the protein in the membrane (*i.e.*, anchored, versus non-anchored, structure, electric charge) and also its interaction with other (membrane) proteins. Additionally, diffusion (of proteins and lipids) can shed additional information on membrane anatomy, such as the existence of domains like lipids rafts. One would expect that the lateral diffusion coefficient (D) of a protein will vary when the protein is bound to another protein, or when the protein is diffusing in a domain whose viscosity is different that of the other membrane regions. However, biophysicists have for over three decades neglected this point due to their reliance on the Saffman-Delbrück (SD) diffusivity model [19] that predicts “weak” dependence of the lateral diffusion coefficient (D) on the radius (R) of the diffusing object and the membrane viscosity (μ) (See Figure 1 and Equation 1 below). Consequently, the implications of this model’s assumptions are that the D values are independent of R and that “all membranes”/membrane domains have the same viscosity! Figure 1 is a schematic representation of a cylindrical object embedded in a liquid membrane, which is surrounded by an aqueous phase on both sides. We will discuss this matter further in Section 3.1.

According to the SD model, the lateral diffusion coefficient (D) is given by the following Equation [19]:

(1) $$D=\frac{k_{b}T}{4\pi \;\mu_{m}\;h}\left(\text{ln}\frac{\mu_{m}\;h}{\mu_{w}\;R}-\gamma \right)$$

where k_b_ is the Boltzmann constant, T is the absolute temperature, h is the membrane thickness, μ_m_ is the membrane viscosity, μ_w_ is the viscosity of the surrounding aqueous phase, R is the radius of the diffusing object, and γ is Euler’s constant (0.5772).

Some widely used experimental methods to measure the lateral diffusion of membrane proteins are; fluorescence recovery after photobleaching (FRAP), fluorescence correlation spectroscopy (FCS), and single particle tracking (SPT). The three methods are described in recent review papers, to which the reader is referred for additional information [20–24]. In addition to FRAP, we briefly discuss the alternative methods of FCS and SPT. Although the three methods may not necessarily provide us with the same diffusion coefficients due to technical issues (see below), it is nonetheless important to mention FCS and SPT while discussing FRAP. Moreover, the majority of biological studies are using these methods to learn more about the structure and or function of the membrane as well as the diffusing species, and not to obtain diffusion coefficients per se. The bulk of this review will focus on some recent experimental results obtained utilizing FRAP. Additionally, we list several recent applications of FRAP to cellular and whole organism systems (*i.e.*, C. *elegans*). These examples illustrate the versatility of this technique in bio-macromolecular studies.

## 2. Materials and Methods

### 2.1. GUVs (Giant Unilamellar Vesicles)

Giant unilamellar vesicles (GUVs) are lipid bilayer vesicles with diameters ranging from about 5 to 300 μm [12]. Owing to their large dimensions (comparable to those of cells) which enable them to be manipulated by micropipettes, GUVs have been utilised as biomimetic membranes in a variety of studies investigating protein and lipid diffusion [25–29]. There are two main methods to prepare GUVs. The first method involves the rehydration of dried lipid films while in the second method, known as electroformation, the rehydration step is done under an electric field. See the following reference for more detailed descriptions of both methods [30].

### 2.2. The Biomimetic Sponge Phase

The tunable biometic sponge (L_3_) phase that has been used in several studies described in this review [25,31] is prepared by mixing a non-ionic surfactant C_12_E_5_ (pentaethylene glycol monododecyl ether), a co-surfactant β-OG (β-octylglucopyranoside), and an aqueous solution (usually the buffer in which the protein of interest is dissolved). The distance between the bilayers (d_w_) is simply tuned by varying the amount of water/aqueous solvent The d_w_ value is measured by either small angle scattering spectroscopy (either SAXS or SANS) or freeze fracture electron microscopy (FFEM), and the addition of membrane proteins at experimentally desired concentrations did not affect the properties of the phase as witnessed by constant d_w_ values in the absence and presence of proteins [31]. A preliminary study on the activity of several transmembrane proteins inside this sponge phase shows that the proteins inserted into the phase retain their biological activity (Picard *et al.*, to be submitted).

### 2.3. FRAP (Fluorescence Recovery after Photobleaching)

**Figure 2.**
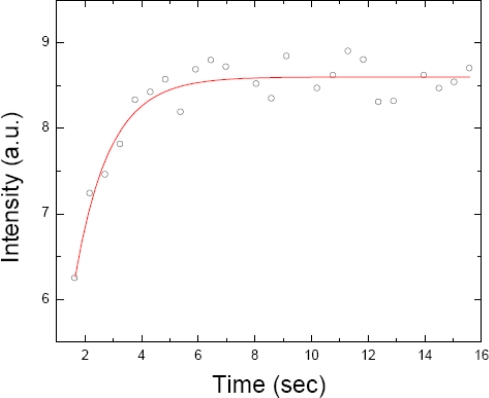
A fluorescence recovery *versus* time curve of fluorescein isothyocyanate (FITC) inside a GUV, as obtained by FRAP (unpublished results of the authors).

As implied in the name, the premise behind FRAP stems from the characteristic of fluorophores to undergo bleaching when exposed to intense light. Briefly, an intense laser light is used to flash a region of the sample (containing fluorescently labelled molecules). After the bleaching, a low intensity laser light is used to follow the recovery of fluorescence caused by the concurrent inward diffusion of neighbouring (non-bleached) molecules into the bleached region and the outward diffusion of the bleached molecules. The analysis of the resulting fluorescence recovery *versus* time curve yields recovery times that are used to obtain diffusion coefficients of the diffusing species. Figure 2 is a fluorescence recovery *versus* time curve of a fluorescent dye (fluorescein) diffusing inside a giant unilamellar vesicle (GUV). In Figure 7 we schematically present several different diffusion scenarios as monitored by FRAP and briefly discussed in this review.

The diffusion coefficient is equal to:

(2) $$D=\frac{r^{2}}{4\tau }$$

where r is the radius of the circular beam, and τ is the time constant obtained from the fit of the curve [32]. The r value needs to be precisely known as any deviation in this value will lead to incorrect D values. This issue is discussed further by Braeckmans *et al.* [33]. The fluorescence recovery curve was fit via the following equation [32,33]:

(3) $$\frac{F_{\mathrm{tot}}\;(t)}{F_{0}}=1+\left\{\sum_{n=1}^{+\infty }\;\left[\frac{\left(-K_{0}\right)}{n!}\;\frac{1}{\sqrt{1+n}}\right]\times \left(1-e^{-2(\tau /t)}\;\left(I_{0}\left(2\frac{\tau }{t}\right)+I_{1}\left(2\frac{\tau }{t}\right)\right)\right)\right\}$$

where F_0_ is the total fluorescence at time zero (*i.e.*, before bleaching), K_0_ is the bleaching parameter, and I_0_ and I_1_ are the modified Bessel functions of the 0th and 1st order respectively [33].

Potential problems with the application of FRAP to cell studies include incomplete fluorescence recovery due to obstruction of diffusion produced by percolation networks [34,35], as well as the need to measure the beam profile [36] (see above). Additionally, when FRAP is utilized for cellular studies, the laser beam is focused on a spot of a fairly small area therefore interrogating a rather limited number of fluorophores and significantly decreasing the signal to noise ratio [37]. Some further problems associated with FRAP may include the possibility of photoinduced crosslinking of labelled proteins (leading to lower D values), as well as a local temperature increase (which may also affect D values). That typical illumination intensities and times in FRAP experiments do not lead to significant photodamage, was demonstrated by a set of eloquent experiments performed by Jacobson *et al.* [38]. The authors clearly confirmed that multiple bleaching of the same spot did not lead to different D values, and that the addition of free radical quenchers did not affect the measured D values [38]. Axelrod has shown that the illumination induced temperature increase (in a FRAP experiment) does not exceed 0.3 °C [39].

While FRAP has been used utilized since the mid seventies to study the dynamics inside cells and cell membranes [40–44], the method has regained popularity with the advent of GFP (green fluorescent protein) technology less than two decades ago [20,45]. The suitability of GFP for cellular studies stems from its following properties; it is stable, non-toxic to the cell, does not bleach significantly at low light intensities, does not seem to be damaging to the cell after undergoing irreversible photobleaching, it can be readily expressed in several cell types where it is fused to a particular protein, and the fusion of GFP to a protein of interest does not affect the function and location of the protein being investigated [45–49]. Thus GFP behaves like a “non-invasive” [48] extrinsic fluorophore, and it has been shown to be more stable than fluorescein isothyocynate (FITC), a widely used fluorophore [49].

### 2.4. FRAPP (Fluorescence Recovery after Pattern Photobleaching)

**Figure 3.**
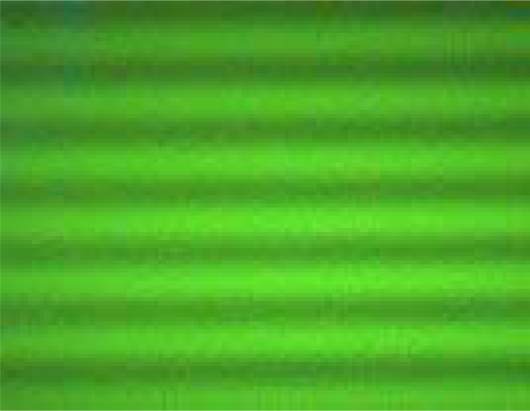
A fluorescence gradient/fringe pattern picture of an FITC sample. The interfringe distance corresponds to 17 μm.

This method has been devised in 1982 in order to ameliorate some disadvantages associated with classical FRAP [36]. Briefly, FRAPP utilizes two laser beams intersecting precisely in the focal plane of a microscope and thus creating a fluorescent fringe pattern gradient [25,36]. Figure 3 is a representative fringe-pattern picture of a fluorescent dye, FITC.

**Figure 4.**
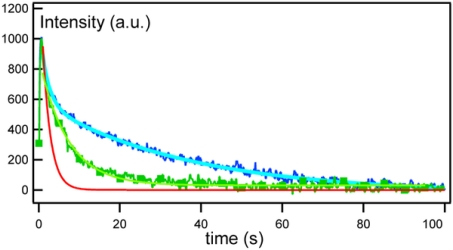
FRAPP curves of biotinylated transmembrane peptide (B-L_12_) streptavidin (S) complexes, ratio r = [B]/[L_12_]; where r = 0 (solid red line), r = 0.2 (green squares), and r = 1.9 (blue solid line). From [31], by permission from PLoS ONE.

Several advantages of the technique include; high sensitivity, the ability to measure D values over a wide range of distances and thus to check for Brownian motion of the diffusing species, and also the ability to discern the presence of differently diffusing species by examining the recovery signal curves [25,31,36]. Figure 4 shows representative FRAPP fluorescence recovery curves obtained by Reffay *et al.* [31]. Each curve corresponds to a different biotinylated transmembrane peptide (B-L_12_) streptavidin (S) ratio r = [B]/[L_12_]; where r = 0 (solid red line), r = 0.2 (green squares), and r = 1.9 (blue solid line), which were fitted by a single, double, and triple exponential, respectively [31]. The results clearly demonstrate that FRAPP can identify the presence of several diffusing species by the nature of the fit of fluorescence recovery curves, whereby a single exponential corresponds to a single diffusing species, and multiple exponentials to several diffusing species.

A FRAPP experiment is performed at several (e.g., 4–5) interfringe distances (i) and a plot of τ^−1^ (where τ is the time constant obtained from the fit of a FRAPP curve, usually an average value of about 10 runs) *versus* i^−1^ is plotted. A linear plot going through the origin is indicative of Brownian diffusion, with the slope being equal to the diffusion coefficient (D) via the classical relation [36]:

(4) $$D=\frac{i^{2}}{4\pi^{2}\;\tau }$$

**Figure 5.**
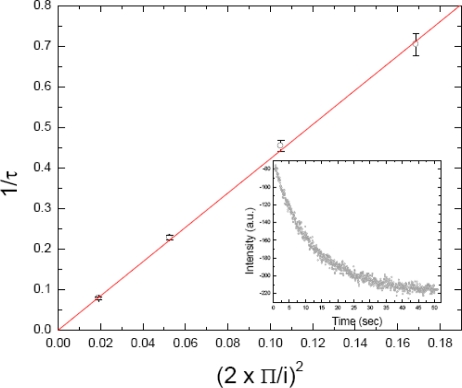
A τ^−1^ *versus* i^−1^ plot for the diffusion of MexA protein inside a sponge phase. The diffusion coefficient corresponds to 4.2 μm^2^/s. The insert is a curve of the decay of fluorescence contrast between bleached and non-bleached region (see Figure 3), which corresponds to fluorescence recovery brought about by the diffusion of the non-bleached species into the bleached area [36,50,51].

Deviations from this are indicative of non Brownian diffusion [36]. Figure 5 is a τ^−1^ *versus* i^−1^ plot for the diffusion of MexA protein inside a biomimetic sponge phase, the D value corresponds to 4.2 μm^2^/s (unpublished results of the authors). The insert of Figure 5 is a fluorescence recovery curve of MexA at an interfringe distance of 45.5 μm (see below).

As mentioned above, FRAP has several drawbacks, especially when applied to cellular studies, that can be overcome by FRAPP. It is of no surprise that the latter method has been applied to study protein diffusion inside cells. Munnelly *et al.* [37] have directly compared FRAP and FRAPP by studying the diffusion of a major histocompatibilty complex (MHC) class II antigen in M12.C3.F6 cells. In addition to the ability to interrogate the whole cell, the reported advantages of FRAPP included a 100 fold improved fluorescence signal for this particular system, hence an improved signal to noise ratio (see Figure 5 of the reference). While the two methods yielded comparable D values as well as percentage mobile fractions, the reproducibility of D values obtained by FRAPP was much better than those obtained by FRAP, and the authors suggested that the former method can be useful in identifying cell-cell variation in protein mobility [37].

## 3. Some Recent Studies

### 3.1. Lateral Diffusion of Membrane Proteins

Gambin and collaborators [25] have used FRAPP to obtain diffusion coefficients of various peptides and transmembrane proteins, inserted into GUVs and a biomimetic (non-supported) sponge phase of tunable thickness, in order to assess the validity of the Saffman-Delbrück (SD) diffusivity model. As mentioned earlier, the SD model predicts that the lateral diffusion coefficient of a protein will be weakly dependent on its radius (R) [19]. By plotting the D values of peptides and transmembrane proteins from their study as well as those obtained by other authors [52], it was revealed that D was inversely proportional to R (D α 1/R) [25], contrary to the SD model that predicts a logarithmic dependence of D with R (D α ln 1/R). Several recent theoretical works have been concerned with this matter. Naji *et al.* [53] proposed that the deviation from the SD model was due to membrane-protein induced perturbation of the membrane, which in turn affected the mobility of the complex leading to the D α 1/R dependence. Utilizing coarse-grained simulations, Guigas and Weiss have argued that the D α 1/R dependence occurs beyond certain critical R values [54,55], which are significantly greater than those for transmembrane proteins, and thus the lateral diffusion of transmembrane proteins should follow the SD model. It should be mentioned that the two coarse-grained studies [54,55] have reported rather high solvent viscosities, as compared to those of the membranes, and this may have adversely affected the simulations. Interestingly, two FCS studies examining the diffusion of transmembrane proteins in GUVs published in 2009 have yielded conflicting results with regards to the dependence of D on R [26,27]. Kriegsmann and colleagues [27] illustrated that the diffusion coefficients of NpSRII (a heptametric helical transmembrane photoreceptor), NpHtrII (a transducer of NpSRII composed of a dimeric helical transmembrane segment, and a cytoplasmic domain), as well as the NpSRII/NpHtrII bound complex display the 1/R dependence, thus being in excellent agreement with the results obtained by Gambin *et al.* [25]. Ramadurai and collaborators [26] have found no D α 1/R dependence when investigating the lateral diffusion of several transmembrane proteins ranging from 1 to 36 transmembrane segments. Thus, their findings were in agreement with the SD model [19], and in contrast to what was reported by the two above-mentioned studies [25,27]. We would like to add that the study by Kriegsman *et al.* [27] utilized dual-focus FCS, while the one by Ramadurai *et al.* [26] relied on single-focus FCS. While in both cases, the proteins were inserted in the same biomimetic media (GUVs), the former study employed the more “advanced” dual-foucs FCS, while the latter relied on the single-focus FCS that has been shown to exhibit under certain condition errors that can lead to flawed D values (see below). Clearly, additional head on studies employing both FCS and FRAP, and perhaps SPT (much like the one performed by Bates *et al.* [56], see below) are warranted in order to test the dependence of D on R for transmembrane proteins. Ideally, the experiments should be very carefully executed and in more or less identical (domain-free) media (*i.e.*, GUVs with the same or similar lipid to protein ratios) in order to minimize potential discrepancies.

### 3.2. Diffusion of Lipids and Membrane Proteins under Membrane Curvature Gradients

Tian and Baumgart [57] have measured the diffusion of DiOC_16_ (a lipid fluorophore) and CTB (Cholera toxin subunit B, a membrane protein used as a tracer in the studies of cellular trafficking) inside a highly curved system of tubular membranes and planar supported bilayers. Tubular membranes were produced by pulling tethers from GUVs. The study was borne out by the hypothesis that membrane curvature may play a role in the segregation of lipids and proteins in cellular processes, such as sorting and trafficking. The distribution of lipids and proteins within respective (curved *versus* non-curved) systems was measured by confocal fluorescence microscopy and revealed that CTB and not lipids sensed the curvature gradient (*i.e.*, CTB was more localised in highly curved tubular membrane as opposed to GUVs). The diffusion coefficients of CTB in supported bilayers were about 8 times smaller than the respective DiOC_16_ values, while on membrane tethers CTB exhibited D values 2 times smaller than those of DiOC_16_. The authors rationalized their finding by suggesting that CTB could be more crowded on flat bilayers as opposed to highly curved membrane tethers [57]. Evidently, further studies should be undertaken to assess the effect of membrane curvature on the lateral diffusion of several membrane proteins in addition to CFTB. Interestingly, a theoretical study by Daniels and Turner [58] proposed a logarithmic dependence of D on the membrane surface tension (σ) for proteins diffusing on a tubular membrane and that one should measure the diffusion of proteins as function of “membrane size” in order to test the validity of the Saffman Delbrück diffusivity models. The membrane size can be varied by varying the tube radius, which in turn can be accomplished by changing the surface tension of the membrane [58]. An experimental verification of authors’ predictions (by measuring D as function of σ) is warranted not only for proteins diffusing in tubular membranes, but for those diffusing in “flat” membranes such as those in GUVs. Perhaps, it is the effect of membrane surface tension in GUVs that plays a role in differing interpretations of the SD model, as reported by several experimental groups [25–27]. We have to clarify that the studies such as the one by Daniels and Turner [58] are based on assumptions that one may apply the SD formalism for small objects. This is an incorrect assumption, and several experimental studies can attest to that [25,52,59].

### 3.3. Hydrophobic Mismatch

As already mentioned, the “insertion” of transemembrane proteins into the bilayer will give rise to the hydrophobic mismatch phenomenon in which the lipids in the bilayer either stretch or compress to accommodate the proteins. If the length of the protein core is greater than that of the bilayer, the lipds will stretch, while they will compress in the reverse case in order to prevent the energetically unfavourable exposure of the hydrophobic region of the protein to the aqueous environment [4]. It ought to be mentioned that for single-helical transmembrane peptides, the matching will be performed by the peptides, which will tilt to fit within the membrane [10,60,61]. Gambin *et al.* [62] have used FRAPP to study the lateral diffusion of transmembrane peptides (poly-leucines) of various lengths, inside GUVs as well as a biomimetic sponge phase, in order to obtain additional information regarding the hydrophobic mismatch. The first experimental system, GUVs of constant bilayer thickness, was utilized to study the effect of peptide length on the phenomenon of hydrophobic mismatch. The peptide L_18_ exhibited the highest mobility, as witnessed by its highest D value. The hydrophobic length of L_18_ matches the membrane thickness of the GUVs, and as expected it had the highest diffusion coefficient. Not surprisingly, the mobility of L_24_ was reduced by about 30% as compared to that of L_18_. L_24_ is tilted in the membrane as confirmed by ATR-FTIR (attenuated total reflectance-Fourier transform infra red spectroscopy) experiments. Unexpected results were obtained for L_12_ whose D value mirrored that of L_18_. The results were rationalized by the fact that the peptide was simultaneously pinching the membrane while being anchored in it. The second system consisted of a biomimetic, tunable sponge phase whose bilayer thickness was varied by the addition of a hydrophobic solvent. The measurements of diffusion coefficients as a function of membrane thickness revealed a similar pattern for all three peptides, where the D values exhibited a maximum at a membrane thickness corresponding to their hydrophobic length. The authors clearly demonstrated the sensitivity of D values to the hydrophobic mismatch, and based on the D values were able to calculate the tilt angles of peptides [62].

### 3.4. Membrane Protein Association

**Figure 6.**
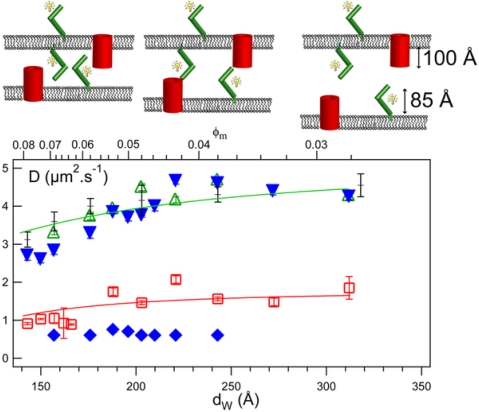
MexA-OprM interactions as a function of bilayer separation distance (d_w_). The top panel is a schematic representation of the interactions between the proteins, with the barrels representing OprM and boomerang-like sticks representing MexA. Each schematic in the upper panel corresponds to proposed interactions between the two proteins as a function of d_w_ where the middle one is representative of interactions at ∼200Ǻ. The bottom panel is a plot of diffusion coefficients as a function of bilayer distance. The symbols correspondence is as follows; green (empty) triangles pointing up; FITC-labelled MexA alone, blue (full) triangles pointing down; non-bound MexA in a sample containing OprM trimers, black crosses; FITC-C_16_ (a fluorescently labelled SOPC lipid) alone, red squares; OprM alone, blue (full) diamonds; MexA bound to OprM. From [31], by permission from PLoS ONE.

Reffay *et al.* [31] have used FRAPP to study the interactions between MexA (a periplasmic protein) and OprM (a transmembrane protein), of the MexA-MexB-OprM efflux pump in *Pseudomonas aeruginosa.* The proteins were inserted into a “tunable” biomimetic sponge phase. Tunable refers to the ability to tune the bilayer thickness as well as to adjust the distance between adjacent bilayers (d_w_), and hence probe the nature of the interaction. The elegance of this system lies in the fact that it is well suited to study lateral membrane-protein interactions, as well as those occurring between proteins inserted into opposing membranes, such as those of a protein complex spanning two membranes. Additionally, the sponge phase is not supported (a potential issue when measuring diffusion of transmembrane proteins) and it does not affect the structure and function of membrane proteins, as witnessed by retained activity of several transmembrane proteins in the sponge phase (Picard *et al.*, to be submitted). Reffay and colleagues have looked at the diffusion inside a sponge phase of MexA, OprM, as well as that of MexA in access of OprM, as a function of bilayer distance ranging from 90–320 angstroms [31]. Figure 6 is a schematic representation of the interactions between MexA and OprM as well as a plot of the D values as a function of bilayer distance. Based on the diffusion *versus* membrane distance, they have determined the following; MexA is anchored to bilayers (as the diffusion of a soluble mutant MexA protein was about ten times greater than that of the wild type protein), OprM is embedded in the membrane (with a lower D value than that of MexA), and that MexA and OprM do not interact laterally but form a complex when they are in opposing membranes with the population of the MexA-OprM complex being at its maximum at ∼200 Å, which corresponds to the periplasmic thickness of *Pseudomonas aeruginosa* [31]. By monitoring the fluorescence intensity, that is the intensity of the signal corresponding to a particular D value of either the free MexA protein or when it is bound to OprM, as a function of molar ratio, the authors were able to compute the MexA/OprM stoichiometry of 2 at pH 7.5. Furthermore, they have shown that this stoichiometry was pH dependent, going from 2 to 6 as pH decreased from 7.5 to 5.5 [31]. This study eloquently demonstrated the versatility of FRAP(P) as a tool for membrane protein studies.

We would like to mention that all the subsequent studies discussed below were performed using classical FRAP.

### 3.5. Thylakoid Membrane Proteins

Kirchhoff *et al.* [63] have studied the diffusion of grana thylakoid membrane chlorophyll-protein complexes from isolated spinach (*Spinacia oleracea*) grana membranes. Grana are thylakoid membrane domains (resembling a stack of coins or discs) and present some of the most crowded membranes in nature with around 70% to 80% of the membrane occupied by proteins [64]. Clearly, one would expect proteins to diffuse slowly in such an environment. Accordingly, the authors have discovered that around 75% of chlorophyll-protein complexes are immobile during measured time-scales of up to 9 min, while the reminder diffused rather rapidly with the D value of ∼0.005 μm^2^/s [63]. This “rapidly” diffusing population was rationalised to be composed of mobile proteins, which exchange between the grana and stroma lamellae [63].

The first study examining the diffusion, and hence “mobility”, of a single thylakoid membrane protein was performed by Vladimirou and colleagues [65]. They have studied Hcf106, a single transmembrane protein, and reported that there was effectively no fluorescence recovery over periods up to 3 min, demonstrating that the protein is either immobile or its diffusion is strictly restricted within certain domains [65]. It appears that Hcf106 is one of those immobile thylakoid membrane proteins, which constitute around 75% of chlorophyll-protein complexes [63]. Evidently, it would be interesting to study a thylakoid membrane protein from the “mobile” group comprising 1/4 of chlorophyll-protein complexes.

### 3.6. Green Fluorescent Protein (GFP) Diffusion in Escherichia Coli

The authors utilized FRAP to study the diffusion of GFP inside elongated *E. coli* cells [66]. More specifically, GFP’s diffusion was investigated in three environments inside the cell; in the plasma membrane (where GFP was fused to TatA, an integral *E. coli* protein), in the cytoplasm, and in the periplasm, and the following respective D values were reported; 0.13 ± 0.03 μm^2^/s, 9.0 ± 2.1 μm^2^/s, and 2.6 ± 1.2 μm^2^/s [66]. While the D value measured in the plasma membrane was the first reported value for the lateral diffusion of a bacterial plasma membrane protein *in vivo,* the D values obtained for GFP diffusion in the cytoplasm and the periplasm were comparable confirming the similar “fluid” structure of the two compartments. This work clearly demonstrated that diffusion of a protein inside various cellular compartments is a good indicator of comparative cellular structure.

### 3.7. Polyglutamine Pathogenesis in Caenorhabditis elegans and Disassembly of β-Amyloids

**Figure 7.**
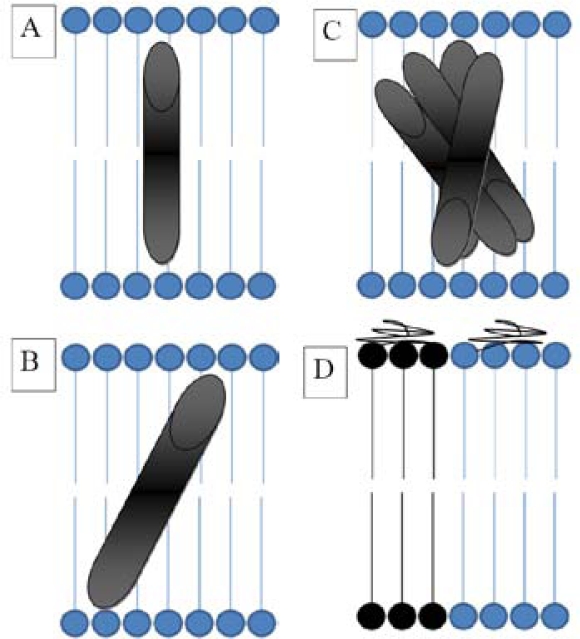
The author’s rendition of a bilayer membrane under various conditions. A.; a protein diffusing under “normal” conditions will exhibit a characteristic diffusion coefficient (D) value, B; a protein under the hydrophobic mismatch will tilt and consequently exhibit a lower D value than the corresponding protein in A, C; Protein aggregates, which will diffuse more slowly and hence have lower D values than a non aggregated protein in A, and D; the effect of domains, such as lipid rafts, will affect the diffusion of adsorbed DNA causing it to have a different D value when diffusing in the domain regions. The panel C of the figure illustrates protein aggregates, and we refer to the diffusion of β-amyloid (Aβ) peptide aggregates as reported in the study by Edwin *et al.* [70]. Assuming that the panel A represents the monomeric Aβ peptide at pH 7.4 with a D value of 155 μm^2^/s, the panel C corresponds to aggregates at pH 2.7 with a D value of ∼3.5 μm^2^/s [70]. The panel D illustrates the diffusion of membrane-adsorbed DNA oligonucleotides in different membrane lipid domains (black and blue). After Athmakuri *et al.* [71]. D values will depend on the area of the membrane. The authors report a D value of 1.67 μm^2^/s under conditions where DSPC membrane composition was 10%, while the diffusion coefficient decreased to 0.14 μm^2^/s when DSPC % was equal to 50. The presence of DSPC domains resulted in decreased diffusivity of adsorbed DNA oligonucleotides [71].

*C. elegans* (a roundworm) has been used as a model organism to study polyglutamine expansions, a neurodegenerative disorder characterized by polyglutamine aggregates and seen in neurodegenerative diseases such as Huntington’s [67,68]. The authors have fused several polyglutamine (Q) proteins to the yellow fluorescent protein (YFP) and measured their diffusion in live, anesthetised organisms. While it is not unreasonable to expect that D values will show some variation according to the length of the Q residues, it was demonstrated that in the muscle cells of *C. elegans,* the diffusion of the Q0:YFP species was rapid, while that of the Q40:YFP did not exhibit recovery after photobleaching indicating that immobile aggregates were formed [67], consistent with length dependent polyglutamine aggregate formation [69]. Unfortunately, the authors did not report diffusion coefficient values, just relative fluorescence recovery times. Figure 7B is a schematic representation of a protein under the hydrophobic mismatch, while Figure 7A shows a corresponding non-mismatched protein. In Figure 7C we illustrate a protein aggregate complex, as compared to the corresponding, non-aggregating, protein under “normal” conditions in Figure 7A.

Edwin *et al.* [70] have used FRAP to study the reversibility of β-amyloid (Aβ) peptide aggregate formation. The Aβ peptide is believed to be the cause of the Alzheimer’s disease pathogenesis, brought about by the conversion of random coil or α-helix peptides to β-sheet fibrilar structures [70]. The authors have looked at the effects of pH and added salt on the reversibility of β-amyloid self-association as assessed by FRAP. Evidently, any factor leading to reversibility of this effect would be witnessed by an increase in D values. The peptide is in its monomeric state at pH 7.4, while it forms aggregates at pH 2.7, and its aggregation can be reversed by an increase in pH [70]. It was reported that an increase in pH from 2.7 to 7.4 was accompanied with an increase in D values from ∼3.5 μm^2^/s to ∼155 μm^2^/s (indicative of more rapidly diffusing/non-aggregating species). The estimated radii corresponded to about 1.5 nm at pH 7.4 and about 90 nm at pH 2.7. Obviously, these results are not in agreement with the SD model and are more adequately explained by the D α 1/R model proposed by Gambin *et al.* [25]. The addition of calcium chloride up to 15 mM promoted aggregation (decrease in D values) and subsequent addition of CaCl_2_ up to 25 mM suppressed aggregation (increase in D values). The effect of this salt has been examined by the investigators because of calcium’s involvement in amyloid diseases [70].

### 3.8. Lateral Diffusion of DNA and the Effect of Lipid Domains/Rafts

Now we turn our attention to the lateral diffusion of adsorbed DNA onto lipid bilayer membranes. Athmakuri *et al.* [71] have studied the lateral diffusion of single-stranded DNA oligonucleotides adsorbed onto supported bilayers of varying membrane heterogeneity (gel DSPC domains in a DOTAP continuous/fluid phase). There are two interesting observations that were reported in this work: 1) Diffusion coefficients of fluorescently-labelled DNA decreased as a function of increasing DSPC %, indicating that the lateral mobility of DNA was retarded with an increase in gel phase/membrane heterogeneity, and 2) The diffusion of 1% NBD-PE (a fluorescent lipid dye) in the presence of non-labelled DNA in the bilayers ranging from 10%–50% mole fraction of DSPC exhibited nearly identical diffusion coefficients to DNA, indicating that the presence of lipd rafts affected the diffusion of fluorescently-labelled lipids much the same way it affected the diffusion of membrane adsorbed DNA oligonucleotides. Furthermore, the above experiments were repeated, but this time another cationic lipid DMTAP, was used instead of DOATP, in order to further examine the effects of membrane heterogeneity/lipd domains on DNA diffusion. Once again, it was seen that the D values of DNA decreased as a function of increasing DSPC mole fraction, and the rate of diffusion of DNA was similar to that of NBD-PE. The take home message of this study was that the diffusion of membrane-adsorbed DNA was restricted by increasing lipid domains presence, and the lateral diffusion of lipids was also affected by these domains [71]. Figure 7 D is a schematic representation of adsorbed DNA in the presence of domains (black circles), as compared to that when little or no domains are present (blue circles). As reported in the study, the presence of domains will result in a decrease in the diffusion coefficient of the adsorbed DNA.

### 3.9. Membrane Confinement and Fluidity

Although this review has focused on the diffusion inside membranes, an interesting recent study has examined the diffusion inside the water layers of artificial or model membranes. Moreau and colleagues [72] have investigated the effects of membrane confinement on the diffusion of two fluorescent dyes (fluorescein and rhodamine) and a fluorescently labelled protein, bovine serum albumin (BSA), inside the aqueous layers of two lyotropic lamellar phases, an ionic AOT/NaCl/H_2_O and a non ionic C_12_E_5_/hexanol/H_2_O phase. The confinement was achieved by “swelling” the membranes, and the separation distance (d_w_) between the membranes (which essentially corresponds to the thickness of the aqueous layer) was varied from ∼20 Ǻ to 500 Ǻ. The authors reported that for both the ionic and non-ionic lamellar phases there exists a first (dilute) regime, where the D values are quasi-linearly proportional to 1/d_w_. In fact, in this regime, the diffusion coefficient values at large d_w_ distances approach those of the probes in bulk water, indicating that the diffusing species do not interact with the surfactant bilayers. The decrease of D values with a decrease in inter-membrane distance was due to the confinement of the probes within the bilayers. Very much similar effect has been observed by Reffay *et al.* [31], were the D values decreased with a decrease in d_w_.

Interestingly, for the non-ionic lamellar phase a second (confined) regime was observed (at low d_w_ values) following the first (dilute) one. Here the diffusion coefficients reach an almost constant, non zero, values at d_w_ values roughly corresponding to and smaller than the diameter of the diffusing particle. The D values in this regime are reported to be on the same order as those of the probes inserted inside the bilayers of the same membranes [72].

## 4. Comparison with Some Alternative Methods

### 4.1. FCS (Fluorescence Correlation Spectroscopy)

This method is based on fluctuations of fluorescence intensity, as fluorescently labelled molecules pass through a (minute sample) volume being observed. These fluctuations are analyzed via time dependent correlation functions yielding diffusion coefficients. Briefly, the autocorrelation curve is obtained from the fluorescence intensity trace as follows [73,74]:

(5) $$G_{\left(\tau \right)}=\frac{\langle \delta F\;(t)\;\;\delta F\;(t+\tau )\rangle }{{\langle F\;(t)\rangle }^{2}}$$

where G is the autocorrelation function, F(t) is the fluorescence intensity as a function of time, τ is the correlation time, and the angular brackets refer to time averaging. For two-dimensional Brownian diffusion in the membrane, the autocorrelation function is expressed as [73,74]:

(6) $$G_{\left(\tau \right)}=\frac{1}{N}{\left(1+\frac{\tau }{\tau_{D}}\right)}^{-1}$$

where N is the average number of fluorescent particles in the detection volume, and τ_D_ is the diffusion time. The diffusion coefficient (D) is as follows [73,74]:

(7) $$D=\frac{w^{2}}{4\tau_{D}}$$

where w is the waist of the focused laser beam. The waist of the focused beam should be precisely calibrated, in order to avoid erroneous D values. This is analogous to the need to precisely measure the laser profile beam in FRAP experiments, as mentioned in Section 2.3.

Potential setbacks of this method include the need for strict vertical positioning, laser beam geometry, as well as “high” fluorophore concentrations, and “slow” diffusion [73,75]. High fluorophore concentrations affect the fluorescence fluctuations of the diffusing fluorophores, while slow diffusion times require longer measurement times, which in turn can affect the stability of the detection volume [73]. Additionally, one-photon FCS has been shown to exhibit under certain conditions a non-Gaussian observation volume, which can alter the diffusion correlation function, thus leading to flawed D values [75,76]. The use of two-photon FCS can circumvent the problem of a non-Gaussian observation volume [76]. FCS has been employed since the early seventies [77–80], gaining popularity in biophysical studies in the nineties [74], and it has been used to study diffusion of in both artificial and cellular membranes. There are several excellent reviews on FCS to which one is referred [73–75,80,81].

### 4.2. SPT (Single Particle Tracking)

Unlike FRAP and FCS, where one follows the dynamics of an ensemble of macromolecules, SPT follows the dynamics of individual molecules (tagged with latex beads, colloidal gold, or fluorophores), whose trajectories are monitored by computer assisted video microscopy. It has been illustrated that SPT can yield erroneous diffusion coefficients when inappropriate sampling frequencies are used, and that noise can lead to errors in accurately finding the actual particle position, which consequently leads to apparent subdiffusion [24,82]. The SPT method has been used to study cell membrane proteins since the early eighties [34,83] and there are several review papers for further information on the method [24,34,84].

We conclude this section by discussing a study that has compared results obtained with FRAP, ICS (image correlation spectroscopy, an analogue of FCS), and SPT. Bates *et al.* [56] have used FRAP, ICS, and SPT to study the lateral mobility and hence diffusion of the cystic fibrosis conductance regulator (CFTR) channel in baby hamster kidney cells. While all three methods detected the presence of significant immobile species of the channel, the diffusion coefficients were shown to be method dependent. The ones obtained using FRAP were around four times faster than the corresponding ICS measurement, while the SPT ones were about two times faster than those obtained by FRAP. SPT explicitly showed the presence of transiently confined CFTR species, and computer simulations revealed that in the absence of confinement areas the D values obtained by FRAP and ICS were similar [56]. The two-fold faster D value measured by SPT (*versus* FRAP) was due to the fact that the value was measured using the mean square displacement (MSD) of the protein when it was outside the confinement areas, and effectively the value corresponded to that of the “rapidly” diffusing proteins [56]. This study illustrates that care should be taken when comparing diffusion coefficients obtained by different methods.

## 5. Conclusions and Future Perspectives

In this review, we have provided ample evidence that FRAP is a versatile and rather easily implementable technique to study the diffusion, and obtain further information on the associations of biopolymers both *in vivo* and *in vitro*. More specifically FRAPP possesses several advantages to classical FRAP as well as complementary methods of FCS and SPT. It is easy to implement, it is “less noisy”, it can astutely differentiate the presence of several diffusing species, and can easily discern Brownian diffusions.

For future perspectives it would also be important to perform further experiments in order to estimate for which “cut-off” R values the SD model is not valid. Care should be taken when conducting studies in order to obtain diffusion coefficients, as it has been illustrated that one can find a different D value based on the method utilized (*i.e.*, single-focus *versus* dual-focus FCS, FRAP *versus* FCS), and this may be caused by insufficient experimental familiarity with one of the methods. If such studies are performed, they should be executed ideally under identical sample conditions. Some further experiments using FRAPP could be performed on thylakoid membrane proteins in order to discern the “mobile” species. It would be interesting to investigate the effects of membrane curvature on the diffusion of a variety of membrane proteins.

## Abbreviations:

FRAP: fluorescence recovery after photobleaching
FRAPP: fluorescence recovery after fringe-pattern photobleaching
FCS: fluorescence correlation spectroscopy
SPT: single particle tracking
D: diffusion coefficient
R: radius
SD: Saffman Delbrück
GUV: giant unilamellar vesicle
FFEM: freeze fracture electron microscopy
SAXS: small angle X-ray scattering
SANS: small angle neutron scattering
FITC: fluorescein isothyocyanate
MHC: major histocompatibility complex
DiOC_16_: 3.3′-dihexadecyloxacarbocyanine iodide
CTB: cholera toxin subunit B
σ: surface tension
ATR-FTIR: attenuated total reflectance-Fourier transform infra red
L_x_: a poly-leucine peptide where x is the number of leucine residues
d_w_: intermembrane distance
GFP: green fluorescent protein,
Q: glutamine
YFP: yellow fluorescent protein
Aβ: β-amyloid peptide
DSPC: distearoylphosphatidylcholine
DOATP: 1,2-dioleoyl-3-trimethylammonium propane
NBD-PE: *N*-(7-nitrobenz-2-oxa-1,3-diazol-4-yl)-1,2-dihexadecanoyl-sn-glycero-3-phospho-ethanolamine
BSA: bovine serum albumin
C_12_E_5_: pentaethylene glycol monododecyl ether AOT/ aerosol OT - sodium 1,4-bis(2-ethyl-hexoxy)-1,4-dioxobutane-2-sulfonate
ICS: image correlation spectroscopy
CFTR: cystic fibrosis conductance regulator
